# Identification of a dietary pattern associated with greater cardiometabolic risk in adolescence

**DOI:** 10.1016/j.numecd.2015.04.007

**Published:** 2015-07

**Authors:** G. Appannah, G.K. Pot, R.C. Huang, W.H. Oddy, L.J. Beilin, T.A. Mori, S.A. Jebb, G.L. Ambrosini

**Affiliations:** a Department of Nutrition and Dietetics, Faculty of Medicine and Health Sciences, Universiti Putra Malaysia, Serdang, Malaysia; b Medical Research Council Human Nutrition Research, Cambridge, United Kingdom; c Diabetes and Nutritional Sciences Division, School of Medicine, King's College London, London, United Kingdom; d Telethon Kids Institute, The University of Western Australia, Perth, Western Australia, Australia; e School of Medicine and Pharmacology, The University of Western Australia, Perth, Western Australia, Australia; f Nuffield Department of Primary Care Health Sciences, University of Oxford, United Kingdom; g School of Population Health, The University of Western Australia, Perth, Western Australia, Australia

**Keywords:** Dietary patterns, Energy density, Fibre, Fat, Cardiometabolic risk factors, Adolescents, Raine study, CVD, cardiovascular diseases, RRR, reduced rank regression, DP, dietary pattern, Raine, Western Australian Pregnancy (Raine) Cohort, FFQ, food frequency questionnaire, BMI, body mass index, WC, waist circumference, HOMA, insulin resistance, y, years, CSIRO, Commonwealth Scientific and Industrial Research Organisation, CDC, Center for Disease Control, IOTF, International Obesity Task Force, HDL-C, high density lipoprotein cholesterol, LDL-C, low density lipoprotein cholesterol, PWC-170, physical working capacity 170, PCA, principal component analysis

## Abstract

**Background and aims:**

Energy dense, high fat, low fibre diets may contribute to obesity in young people, however their relationships with other cardiometabolic risk factors are unclear. We examined associations between an ‘energy-dense, high-fat and low-fibre’ dietary pattern (DP) and cardiometabolic risk factors, and the tracking of this DP in adolescence.

**Methods and results:**

Data was sourced from participants in the Western Australian Pregnancy (Raine) Cohort Study. At 14 and 17 y, dietary intake, anthropometric and biochemical data were measured and z-scores for an ‘energy dense, high fat and low fibre’ DP were estimated using reduced rank regression (RRR). Associations between DP z-scores and cardiometabolic risk factors were examined using regression models. Tracking of DP z-scores was assessed using Pearson's correlation coefficient.

A 1 SD unit increase in DP z-score between 14 and 17 y was associated with a 20% greater odds of high metabolic risk (95% CI: 1.01, 1.41) and a 0.04 mmol/L higher fasting glucose in boys (95% CI: 0.01, 0.08); a 28% greater odds of a high-waist circumference (95% CI: 1.00, 1.63) in girls. An increase of 3% and 4% was observed for insulin and HOMA (95% CI: 1%, 7%), respectively, in boys and girls, for every 1 SD increase in DP z-score and independently of BMI. The DP showed moderate tracking between 14 and 17 y of age (*r* = 0.51 for boys, *r* = 0.45 for girls).

**Conclusion:**

An ‘energy dense, high fat, low fibre’ DP is positively associated with cardiometabolic risk factors and tends to persist throughout adolescence.

## Highlights

• Relatively little is known about diet and the early development of cardiometabolic risk factors in young people.
• Few studies have examined how dietary patterns track during childhood.
• An energy-dense, high fat, low fibre dietary pattern is associated with poorer cardiometabolic health in adolescence.
• This type of dietary pattern tracks moderately during adolescence.
• The early establishment of healthy eating habits appears important for reducing later cardiometabolic risk.

## Introduction

There is growing evidence that cardiometabolic risk factors, namely obesity, high systolic blood pressure, dyslipidaemia, impaired glucose tolerance and vascular abnormalities, develop early in life and track during growth and development and into adulthood [1], [2], [3]. Early identification and understanding of these risk factors are essential, so that appropriate interventions can be targeted to children and adolescents to minimise the risk of developing cardiovascular diseases (CVD) in later life.

Diet is a modifiable risk factor for CVD in adulthood and is therefore, likely to be important for early CVD risk factors in childhood [4], [5]. A number of studies have examined prospective relationships between single food groups and cardiometabolic risk factors in children and adolescents [6], [7], [8]. However, foods and nutrients are not consumed in isolation. Interest in overall dietary patterns (DP) has peaked over the last decade, as these consider all food and nutrient intakes and may account for the cumulative and interactive effects of foods and nutrients eaten together. To date, few studies have examined empirically derived DPs in relation to cardiometabolic risk factors among adolescents [9], [10], [11], [12], [13]. Furthermore, little is known about how DPs track from childhood to adulthood [14], [15]. Understanding how DPs track over the life course may be useful in improving dietary intakes and related health outcomes.

Dietary energy-density, fibre and fat are associated with CVD risk in adults [13], [16], [17], [18] and adiposity in children [12]. In this study, we hypothesised that a DP specifically characterised as energy-dense, high in fat, and low in fibre would be prospectively associated with obesity and other cardiometabolic risk factors, and would track between 14 and 17 years (y) of age in adolescents from the Western Australian Pregnancy (Raine) Study.

## Methods

### Study population

Details of the Raine Study have been described elsewhere [19]. In brief, the original cohort comprised of 2900 pregnant women who were recruited into a trial at King Edward Memorial Hospital (Perth, Western Australia) to examine ultrasound imaging from 1989 to 1991. A total of 2868 babies born to 2804 mothers who remained with the study formed the Raine cohort and were followed up at regular intervals after birth. The present analysis uses data collected at the 14 (n = 1857) and 17 (n = 1709) y follow ups, when comprehensive dietary and cardiometabolic data were collected. Ethical approval for the study was obtained from the ethics committees of King Edward Memorial Hospital and Princess Margaret Hospital for Children. Written informed consent was obtained from all adolescents and their parent or guardian.

### Dietary assessment

A validated semi-quantitative food frequency questionnaire (FFQ) was administered at 14 and 17 y of age to estimate habitual dietary intake over the previous year and has been described elsewhere [20]. Of the 1857 and 1709 adolescents who participated in the 14 and 17 y follow ups, 1611 (87%) and 1009 (60%) completed the (FFQ), respectively. At 14 y of age, the FFQ was completed with assistance from a parent or caregiver. The FFQ collected information on usual frequency of consumption and serving sizes (in household units) of 227 food and beverages; at 17 y of age the FFQ included alcoholic beverages, increasing the number of items to 232. The selected frequency of consumption for each food was converted to a daily intake and linked with Australian Food Composition Tables to calculate average daily nutrient intakes, by the Commonwealth Scientific and Industrial Research Organisation (CSIRO) Australia [21]. Intakes of all food and beverages were then collapsed into 46 (47 at 17 y, including alcohol) predefined food groups based on nutrient profiles or culinary usage, and their hypothesized contribution to diet-disease relationships (Supplementary Table 1). All FFQs were checked by a research nurse and, missing and unclear responses were corrected with the adolescent at the time of their physical assessment.

Dietary misreporting, particularly under-reporting is common among adolescents [22]. Dietary misreporting was estimated using a standardised equation at 14 and 17 y of age, as previously described [22]. Rather than excluding dietary misreporters, a categorical variable indicating plausible, under- and over-reporting was included as a potential confounder in statistical models.

### Dietary patterns

Reduced rank regression (RRR) is a data-dimension reduction technique that identifies DPs that are potentially relevant for the aetiology of disease by including *a-priori* knowledge e.g. biomarkers or nutrient intakes. Identification and evaluation of an energy-dense, high fat, low fibre DP using RRR at 14 y in this cohort has been previously described [23]. Briefly, the RRR model included intakes of all predefined food groups (Supplementary Table 1) as predictor variables, and intakes of dietary energy density (DED) from food, the proportion of total energy from total fat (%E fat) and fibre density as response variables. These three response variables were chosen because of their links with obesity and other cardiometabolic risk factors [12], [24]. DED was calculated by dividing total food energy (kJ) by total food weight in grams (g), excluding beverages. Beverages were excluded from the calculation of DED because they may disproportionately influence total dietary energy density values [25]. Beverages were defined as high and low-fat milk, sugar-sweetened beverages, low-energy beverages, fruit juice, hot and powdered drinks, water and alcoholic drinks. However, to account for their potential contribution to the DP, beverages were included as a food group (Supplementary Table 1) in the RRR analysis. Fibre density was expressed as absolute intake of fibre (g/day) divided by total daily energy intake (MJ). Percentage energy from total fat was calculated by dividing total energy intake from fat (kJ) by total energy intake (kJ) and then multiplying by 100.

A similar ‘energy dense, high fat, low fibre’ DP was identified at 14 and 17 y of age [23]. This DP was most strongly characterised by high intakes of processed meat, chocolate and confectionery, low-fibre bread, crisps and savoury snacks, fried and roasted potatoes, as signified by their strong positive factor loadings, and low intakes of fresh fruits, vegetables, legumes, high-fibre bread and yogurts (which had the greatest negative loadings) at 14 and 17 y of age [23]. As such, intakes of foods with strong positive factor loadings increase the individual's DP z-score; intakes of foods with the greatest negative loading decrease the DP z-score. Each respondent received a z-score for the DP at 14 and 17 y of age, discriminating how strongly their dietary intake corresponded to the pattern. The DP was also positively correlated with intakes of total, saturated- and monounsaturated fat, retinol, cholesterol and negatively correlated with carbohydrate, protein, fibre, magnesium, potassium, folate, vitamin C, iron, thiamine, vitamin A, calcium, niacin, riboflavin, and zinc [23]. This DP identified from a FFQ showed moderate reliability when compared to a 3-day (d) food record in this cohort at 14 y of age [23]. No important gender differences in this DP were observed.

### Measurement of cardiometabolic risk factors

Height was measured using a Holtain stadiometer without shoes and weight was recorded using Wedderburn digital chair scale with light clothing. BMI z-scores were calculated based on age and gender specific growth charts from the Centre for Disease Control (CDC) [26]. Waist circumference was measured by horizontally positioning a measurement tape across the umbilicus at the smallest girth and the average of two measurements included in the analyses. Overweight and obesity was defined using the International Obesity Task Force (IOTF) cut-offs [27], while ‘high-waist’ and ‘low-waist’ circumference categories were defined by WC equal or greater than 80^th^ and less than 80^th^ percentile, respectively [28].

At both the 14 and 17 y follow ups all eligible adolescents were requested to fast overnight before venepuncture by a home visiting phlebotomist. Fasting serum insulin, glucose, triglycerides, high-density lipoprotein cholesterol (HDL-C) were analysed in the PathWest Laboratories at Royal Perth Hospital using standardised methods detailed previously [29], [30]. Low density lipoprotein cholesterol (LDL-C) was calculated using the Friedewald formula and insulin resistance was estimated using Homeostasis Model Assessment (HOMA) [31], [32].

To avoid using an adult definition in estimating metabolic syndrome in children, a two-step cluster analysis previously created by Huang et al. categorised study adolescents into groups at high or low metabolic risk [30]. At both ages, being classified in the high risk metabolic cluster was associated with a significantly greater BMI, waist circumference, insulin, systolic blood pressure, fasting triglycerides and HOMA, and lower HDL-C, compared with those in the low-risk metabolic cluster [30], [33].

### Covariates

Physical fitness was measured using the Physical Working Capacity 170 test (PWC-170) at each clinic session on an ergometer bicycle. Data on PWC-170 has been reported to be highly correlated with self-reported physical activity in this cohort [34]. Information on adolescents' smoking status was obtained at 14 and 17 years of age using an online questionnaire which asked about the number of cigarettes smoked in the last seven days [35].

### Statistical methods

Continuous variables with a normal distribution were described as mean ± standard deviation (SD) and non-normally distributed continuous variables were described as medians with interquartile ranges (IQR). Normally and non-normally distributed variables were compared at 14 and 17 y of age using independent t-tests and Wilcoxon-Mann-Whitney tests, respectively.

Prospective associations between DP z-scores and cardiometabolic risk factors at 14 and 17 y of age were analysed using generalised estimating equations (GEE) with an exchangeable correlation structure. Continuous outcomes included BMI z-score, waist circumference z-score and biomarker concentrations including glucose, insulin, HOMA, HDL-C, LDL-C and triglycerides. Logarithmic transformation (ln) was applied to insulin, HOMA and triglyceride measurements as they were not normally distributed. The beta coefficients resulting from the regression models for these biomarkers were back-transformed for interpretation. GEE models were also used to examine binary outcomes including: odds of overweight or obesity, the odds of a ‘high-waist’ circumference and odds of high-risk metabolic cluster. Models for both continuous and binary outcomes were adjusted for age, dietary misreporting, smoking status, physical fitness and BMI z-score (where appropriate). Since BMI z-score was included as one of the parameters in the definition of the metabolic cluster, we did not adjust for this in the models where metabolic risk cluster was the outcome. We did not adjust for total energy intake, as this is likely to at least partially mediate relationships between diet and cardiometabolic outcomes, particularly obesity. Furthermore, DED is positively correlated with total energy intake and the inclusion of DED in the DP analysis (as a response variable) renders the DP z-score a function of energy intake. Although self-reported data were collected on pubertal status (Tanner stages of pubic hair development) the response rate to this question was poor. Inclusion of pubertal status as a covariate in the models did not make a substantial difference to the size of associations. Therefore, to maximise the number of participants included in this analyses, pubertal status was not included in final models. Separate analyses were run in boys and girls due to the sex dimorphism and puberty-related differences in growth [36].

Tracking of DP z-scores (or maintenance of the same z-score) between 14 and 17 y of age was assessed using partial Pearson correlation coefficient by adjusting for dietary misreporting. The tracking coefficient ranges from 0 to 1, with 1 indicating perfect tracking and 0 indicating no tracking. Since magnitude of tracking coefficient depends on the duration of follow-ups and measurement errors, there are no universally accepted cut-offs to define low or high tracking. However, we chose a coefficient ≤0.30 as the cut-off point for suggesting low tracking, between 0.30 and 0.59 for moderate tracking and ≥0.60 as high tracking to contrast tracking coefficients in this study.

All analyses were performed using Stata 12 (StataCorp, College Station, Texas). A significant level of *P* < 0.05 was considered statistically significant.

## Results

Characteristics of the Raine Study participants at 14 and 17 y of age are shown in Table 1. Between 14 and 17 y, changes indicative of normal growth and changes in body composition were observed. Statistically significant increases were seen for BMI, waist circumference and waist circumference z-score in girls and boys. However, the prevalence of being overweight or obese decreased among boys. The prevalence of high-waist circumference did not change significantly between the follow ups, but boys (32%) were more likely to have a high waist circumference than girls (28%). The prevalence of boys and girls in the high-risk metabolic cluster was reduced significantly between 14 and 17 y of age.

**Table 1:** Cardiometabolic characteristics of the Raine adolescents at 14 and 17 y of age by sex.

Cardiometabolic risk factors	Girls				P-value	Boys				P-value
	14 y		17 y			14 y		17 y		
	n	Mean (SD)	n	Mean (SD)		n	Mean (SD)	n	Mean (SD)	
BMI z-score^a^	780	0.06 (1.0)	620	0.001 (1.0)	0.897	825	−0.06 (1.0)	631	−0.07 (1.0)	0.857
BMI (kg/m^2^)^b^	780	21.5 (4.1)	620	22.9 (4.3)	<0.001	825	21.1 (4.1)	631	22.6 (4.1)	<0.001
Waist circumference z-score^a^	766	−0.08 (0.9)	592	0.18 (1.0)	<0.001	814	0.07 (1.1)	605	0.46 (1.0)	<0.001
Waist circumference (cm)^b^	766	74.6 (10.1)	592	77.5 (11.4)	<0.001	814	76.3 (11.5)	605	80.5 (10.9)	<0.001
Glucose (mmol/L)^a^	664	4.59 (0.60)	614	4.66 (0.53)	0.038	712	4.66 (0.74)	654	4.89 (0.62)	<0.001
Fasting insulin (mU/L)^b^	664	11.13 (1.57)	614	7.61 (1.95)	<0.001	712	9.87 (1.79)	654	7.03 (1.97)	<0.001
HOMA-IR^b^	664	2.32 (1.67)	614	1.57 (2.01)	<0.001	712	2.14 (1.86)	654	1.51 (2.03)	<0.001
HDL-C (mmol/L)^a^	664	1.43 (0.32)	614	1.38 (0.31)	0.010	712	1.35 (0.31)	654	1.21 (0.24)	<0.001
LDL-C (mmol/L)^a^	664	2.38 (0.61)	614	2.44 (0.67)	0.124	712	2.26 (0.64)	654	2.26 (0.67)	0.983
Triglycerides (mmol/L)^b^	664	0.95 (1.45)	614	0.94 (1.51)	0.550	712	0.88 (1.55)	654	1.03 (1.55)	<0.001
Physical fitness (PWC-170)^a^	640	96.8 (19.4)	526	99.7 (24.5)	0.023	694	124.3 (32.2)	580	154.5 (41.5)	<0.001
	**n**	**%**	**n**	**%**	**P-value**	**n**	**%**	**n**	**%**	**P-value**
Overweight/obese (%)^c^	780	24.5	620	23.7	0.736	825	26.5	631	22.0	0.047
High-waist circumference (%)^c^	766	26.9	592	27.5	0.793	814	32.6	605	32.4	0.950
High-risk metabolic cluster (%)^c^	658	32.2	509	19.4	<0.001	703	26.2	544	16.4	<0.001
Plausible dietary reporters (%)^c^	688	56.1	453	43.3	<0.001	730	71.2	404	62.6	<0.001
Smoker (%)^c^^,^^d^	772	2.1	618	18.3	0.078	810	1.0	605	15.5	0.200

a Data are expressed as mean (SD) and were compared using independent t-test.

b Data are expressed as median (IQR) and were compared using Wilcoxon-Mann-Whitney test by sex and age.

c Data were compared using chi-square test by sex and age.

d Smoking ≥1 cigarette(s) in the past one week; High-risk metabolic cluster is a composite indicator of an overall metabolic risk (23); Overweight/obese as defined by the IOTF criteria (25); High-waist circumference was defined as waist circumference ≥80 cm (26).

Prospective associations between the ‘energy dense, high fat and low fibre’ DP and cardiometabolic risk factors are presented in Table 2, Table 3. A higher DP z-score between 14 and 17 y of age was associated with the odds of being in the high-risk metabolic cluster in boys (OR = 1.19; 95% CI: 1.02, 1.39) and girls (OR = 1.04; 95% CI: 1.00, 1.22). After additional adjustment for physical fitness and smoking status, these associations were strengthened in boys (OR = 1.20; 95% CI: 1.01, 1.41) but attenuated in girls (OR = 1.03; 95% CI: 0.87, 1.22) (Table 2).

**Table 2:** Adjusted odds of cardiometabolic risk factors associated with an ‘energy dense, high fat and low fibre’ DP z-score between 14 and 17 y of age, Raine study.

Cardiometabolic risk factors	Girls			Boys		
	n	Odds ratio	95% CI	n	Odds ratio	95% CI
High-risk metabolic cluster^a^						
Model 1	608	1.04	(1.00, 1.22)	646	1.19	(1.02, 1.39)
Model 2	558	1.03	(0.87, 1.22)	605	1.20	(1.01, 1.41)
Overweight/obese^b^						
Model 1	754	0.90	(0.80, 1.01)	784	1.00	(0.90, 1.01)
Model 2	649	1.02	(0.87, 1.19)	699	1.04	(0.90, 1.20)
High-waist circumference^c^						
Model 1	746	1.00	(0.90, 1.02)	781	1.00	(0.90, 1.01)
Model 2	643	1.13	(0.97, 1.32)	697	1.08	(0.95, 1.22)
Model 3	643	1.28	(1.00, 1.63)	697	1.00	(0.82, 1.22)

Model 1 was adjusted for age and dietary misreporting.

Model 2 was adjusted for age, dietary misreporting, physical fitness and smoking status.

Model 3 was adjusted for age, dietary misreporting, physical fitness, smoking status and BMI z-score.

a Composite indicator of an overall metabolic risk (23).

b Overweight/obese as defined by the IOTF criteria (25).

c High-waist circumference was defined as waist circumference ≥80 cm (26).

**Table 3:** Adjusted prospective associations between an ‘energy dense, high fat and low fibre’ DP and cardiometabolic risk factors between 14 and 17 y of age, Raine study.

Cardiometabolic risk factors	Girls			Boys		
	n	β	95% CI	n	β	95% CI
BMI z-score						
Model 1	754	−0.01	(−0.05, 0.03)	784	0.03	(−0.02, 007)
Model 2	649	−0.01	(−0.05, 0.04)	699	0.03	(−0.01, 0.08)
Waist circumference z-score						
Model 1	746	0.01	(−0.04, 0.06)	781	0.03	(−0.02, 0.07)
Model 2	643	0.02	(−0.03, 0.08)	697	0.04	(−0.02, 0.09)
Model 3	643	0.04	(0.01, 0.07)	697	0.003	(−0.02, 0.03)
Insulin (%)^a^						
Model 1	608	4.0	(1%, 8%)	648	6.0	(3%, 9%)
Model 2	558	3.0	(1%, 7%)	605	5.0	(2%, 9%)
Model 3	558	3.0	(1%, 7%)	605	3.0	(1%, 7%)
HOMA (%)^a^						
Model 1	608	5.0	(2%, 9%)	648	7.0	(3%, 10%)
Model 2	558	4.0	(1%, 8%)	605	5.0	(2%, 10%)
Model 3	558	4.0	(1%, 7%)	605	4.0	(1%, 7%)
Glucose (mmol/L)						
Model 1	608	0.0003	(−0.03, 0.04)	648	0.04	(0.004, 0.08)
Model 2	558	−0.004	(−0.04, 0.03)	605	0.05	(0.01, 0.07)
Model 3	558	−0.01	(−0.04, 0.03)	605	0.04	(0.01, 0.08)
HDL-C (mmol/L)						
Model 1	608	0.02	(0.003, 0.04)	648	−0.01	(−0.02, 0.01)
Model 2	558	0.02	(0.001, 0.04)	605	−0.01	(−0.02, 0.01)
Model 3	558	0.02	(0.002, 0.04)	605	−0.002	(−0.02, 0.01)
LDL-C (mmol/L)						
Model 1	608	0.04	(−0.003, 0.07)	648	0.01	(−0.03, 0.04)
Model 2	558	0.04	(−0.01, 0.08)	605	0.003	(−0.03, 0.04)
Model 3	558	0.04	(−0.01, 0.08)	605	0.001	(−0.04, 0.03)
Triglycerides (%)^a^						
Model 1	608	1.0	(1%, 3%)	648	1.0	(1%, 4%)
Model 2	558	1.0	(0%, 3%)	605	1.0	(0%, 4%)
Model 3	558	1.0	(0%, 3%)	605	1.0	(0%, 3%)

a β coefficient values were back transformed using exponential function; Model 1 was adjusted for age and dietary misreporting; Model 2 was adjusted for age, dietary misreporting, physical fitness and smoking status; Model 3 was adjusted for age, dietary misreporting, physical fitness, smoking status and BMI z-score.

No associations were observed between DP z-score and odds of overweight or obesity or BMI z-score in boys or girls (Table 2, Table 3). However, a higher DP z-score between 14 and 17 y was associated with significantly greater odds of a large waist circumference (OR = 1.28; 95% CI: 1.00, 1.63) and waist circumference z-score (β = 0.04; 95% CI: 0.01, 0.07) among girls, after adjustment for all covariates (Table 2, Table 3).

A higher DP z-score was positively associated with a greater insulin concentration and HOMA in boys and girls (Table 3). In models adjusted for age, dietary misreporting, physical fitness and smoking status, a 1 SD unit increase in DP z-score between 14 and 17 y of age was associated with a 5.0% (95% CI: 2.0%, 9.0%) and 3.0% (95% CI: 1.0%, 7.0%) higher fasting insulin concentration in boys and girls, respectively. These associations were slightly attenuated among boys after adjustment for BMI z-score but remained statistically significant (Table 3). Mean HOMA was increased by 4.0% (95% CI: 1.0%, 7.0%) in boys and girls with every 1 unit SD increase in DP z-score, independent of age, dietary misreporting, physical fitness, smoking status and BMI z-score (Table 3).

A greater DP z-score was associated with a higher fasting glucose in boys (β = 0.04, 95% CI: 0.01, 0.08) after adjustment for all covariates (Table 3). There were no significant relationships observed with fasting glucose in girls. Unexpectedly, among girls, HDL-C concentrations increased significantly on average by 0.02 mmol/L (95% CI: 0.002, 0.04) for every 1 unit SD increase in DP z-score between 14 and 17 y of age, after adjustment for all covariates (Table 3). No association was observed with HDL-C for boys and there were no significant relationships observed between the DP and LDL-C or triglycerides in boys or girls (Table 3).

The tracking correlation coefficient between z-scores for the ‘energy dense, high fat and low fibre’ DP at 14 and 17 y of age was 0.51 (95% CI: 0.43, 0.58) for boys and 0.45 (95% CI: 0.37, 0.52) for girls, indicating moderate tracking of the DP.

## Discussion

In this study we observed that an ‘energy dense, high fat and low fibre’ DP is prospectively associated with unfavourable cardiometabolic risk factors, including higher levels of insulin and insulin resistance, in boys and girls. There was also evidence of a positive association with overall metabolic risk and fasting glucose in boys, and waist circumference in girls, independent of BMI.

The positive relationship between the ‘energy-dense, high fat, low fibre’ DP and unfavourable cardiometabolic indicators in this study may be explained by the nutrient composition of the DP. For example, intake of saturated fat, which is known as dietary predictor of CVD risk [13], [16], [17], was positively correlated (r = 0.26; p < 0.05) with z-scores for this DP [23]. Conversely, the intake of fibre, which is known as a protective factor for CVD [13], [18], had the greatest negative correlation (r = −0.37; p < 0.05) with the DP [23]. A combination of higher intakes of saturated fats as well as lower intakes of dietary fibre may influence insulin and insulin resistance through greater glycaemic and insulinemic responses [37], [38].

While the ‘energy dense, high fat and low fibre’ DP was identified using *a priori* information on obesity, this DP was not associated with BMI z-scores or with overweight or obesity in this cohort. This is in contrast to a large UK pregnancy cohort, in which a very similar ‘energy-dense, high fat, low fibre’ DP identified using RRR was found to be longitudinally associated with increased fat mass and excess adiposity between 7 and 15 y of age [11], [12]. The lack of associations seen in the Raine Study could be due to the use of BMI (rather than fat mass) to measure adiposity, or the response variables included in the DP may have not been most relevant to obesity risk in this cohort. It could be that a DP associated with other response variables e.g. carbohydrates or sugars may predict BMI z-scores or obesity risk in this cohort. Although we attempted to adjust for dietary misreporting, a lack of precision in the FFQ could have also obscured the associations observed between the identified DP and BMI z-scores and obesity. However, the ‘energy dense, high fat and low fibre’ DP identified in this FFQ has been shown to be reproducible in a 3-day food record collected in this cohort [23].

Although reductions in insulin and insulin resistance are typically observed post-puberty, we found positive relationships between insulin, insulin resistance and DP z-scores between 14 and 17 y of age [39]. These positive relationships were independent of BMI, highlighting the potential importance of DPs for cardiometabolic health even among adolescents who are not overweight. Positive relationships between similar ‘unhealthy’ or ‘Western’ DPs and components of metabolic syndrome have been reported independent of BMI in observational studies among adults [40], [41].

In contrast to our hypothesis, a positive relationship was observed between the DP and HDL-C concentrations in girls. Positive associations between ‘unhealthy’ or ‘Western’ DPs and HDL-C have been reported in two studies in adults [42], [43]. This deserves further investigation, or alternatively, it may be a chance finding. The gender differences in associations between the DP and cardiometabolic risk factors may be due to unmeasured factors such as lipid accumulation and hormonal differences between the sexes [44].

We have previously reported positive associations (mostly in girls) between high intakes of sugar sweetened beverages (SSB) and cardiometabolic risk factors in the Raine Study, between 14 and 17 years of age [6]. In these analyses we adjusted for overall DPs (‘Western’ and ‘Healthy’ patterns derived using exploratory factor analysis) to test if SSB were a marker of a poor overall DP. Some associations were attenuated but remained statistically significant, while other associations were no longer statistically significant after adjustment for the DPs indicating that both SSB and other components in the overall DP may be important for cardiometabolic health. As a result of these findings and to take a more holistic look at the whole diet, we used hypothesis-based DPs in the present study to investigate whether dietary energy-density, fat and fibre may be key components of a DP associated with cardiometabolic risk factors. Taken together, we believe the findings of this and our previous study indicate that while SSB intake is a cause for concern, an overall DP that is high in energy-density and fat and low in fibre, also contributes to cardiometabolic risk factors in adolescence.

Few prospective studies have analysed the association between DPs and cardiometabolic risk factors other than obesity in children and adolescents [9], [10], [13]. In a cross-sectional analysis, the exploratory ‘Western’ DP previously identified in the Raine study was found to be positively associated with the high metabolic risk cluster and greater waist circumference and BMI in girls at 14 years of age [10]. In the Young Finns Study, a ‘traditional’ DP characterised by high intakes of potatoes, butter, sausages using PCA between three to 18 y of age was positively associated with total cholesterol, LDL-C, apolipoprotein B, and C-reactive protein (CRP) concentrations 21 y later, in women and men, as well as with systolic blood pressure and insulin concentrations in women [9]. A prospective cohort study in the US concluded that a greater adherence to a ‘meat’ DP (high intakes of red meat, refined grain, and butter) in early adulthood was prospectively associated with early atherosclerotic indicators namely E-selectin and P-selectin over a period of 15 y [13].

Previous studies have reported the tracking of intakes of single nutrients or specific food groups e.g. fruits and vegetables [45], [46], but few have examined the tracking of DPs in adolescents [14], [15]. The energy-dense, high fat, low fibre DP observed in our study showed moderate tracking between 14 and 17 y of age, and was slightly higher in boys. This suggests that adolescents in this study were in general likely to maintain a DP associated with adverse cardiometabolic risk factors. Tracking was slightly weaker among girls, possibly reflecting less stable dietary intakes due to attempts at dieting for weight loss, or dietary restraint, related to body image concerns. This suggests that interventions to establish healthy dietary practices may need to begin early in life.

The strengths of this study include the prospective cohort design, repeated measurements of dietary intake and cardiometabolic risk factors, application of an *a-priori* method to identify a nutrient-specific DP and the ability to control for dietary misreporting. In addition, the use of previously defined metabolic clusters avoided arbitrary definitions for metabolic syndrome in this study and identified all individuals who are at greater metabolic risk. Despite these strengths, there are some limitations. Participants in the Raine Study were mainly Caucasian with the majority from families with medium to high incomes. The participation rate at 14 and 17 y of age was 64.7% and 59.6% of the eligible cohort, respectively. However, these participation rates were considered modest, given the long follow-up period of this pregnancy cohort. Estimated intakes of food groups and the response variables used in the DP analysis may have been affected by measurement and correlated errors inherent to the dietary assessment method. Nevertheless, it has been suggested that the use of nutrient densities (e.g. DED, fibre density and %E fat) can reduce the error linked to the dietary assessment method [47]. Although adjustments were made for several potential confounders in the analyses, residual confounding cannot be ruled out.

## Conclusion

An energy dense, high fat and low fibre DP during adolescence is prospectively associated with a greater overall metabolic risk and with insulin resistance and WC, independent of BMI. This DP demonstrates moderate tracking during adolescence. This highlights the role of DPs in adolescence and the importance of establishing healthy eating habits early in life to reduce later cardiometabolic risk.

## Disclosures

Authors have no conflicts of interest to declare.

## Authorship

GA was responsible for data analyses, data interpretation and primary manuscript writing; GP assisted with data interpretation and manuscript writing; RCH was responsible for the cluster analysis of metabolic syndrome and provided critical review of the manuscript; WHO was a principal investigator for the collection of dietary data from which the data for the current study were taken, and provided critical review of the manuscript; TAM and LJB were responsible for the biochemical data collection and analysis, and provided critical review of the manuscript; SAJ contributed to data interpretation and provided critical review of the manuscript; GLA conceived the analysis, advised on data analysis, data interpretation and provided critical review of the manuscript. All authors have read and approved the final manuscript.

## Funding sources

This work was supported by a program grant from the 10.13039/501100000265Medical Research Council (grant number U105960389) and research grants from the National Health and Medical Research Council of Australia (ID#1022134 (2012–2014)) and the National Heart Foundation of Australia and Beyond Blue Cardiovascular Disease (Grant number G 08P 4036) and Depression Strategic Research Program. GA was supported by a PhD studentship from the Ministry of Higher Education, Malaysia and Universiti Putra Malaysia. WHO, TAM and RCH are funded on NHMRC fellowships. Management funding for the Raine Study was provided by the University of Western Australia, the Telethon Kids Institute, the Raine Medical Research Foundation, the Faculty of Medicine, Dentistry and Health Sciences of the University of Western Australia, the Women's and Infants Research Foundation and Curtin University.
