# Supplementary material for: Identification of a dietary pattern associated with greater cardiometabolic risk in adolescence
Source: Nutr Metab Cardiovasc Dis. 2015 Jul;25(7):643–50. doi: 10.1016/j.numecd.2015.04.007 (PMC4510146; doi:10.1016/j.numecd.2015.04.007)
Supplement: Supplementary file 1 [file mmc1.docx]

Table 1: Cambridge Food Groups used in the RRR analysis

| # | Food groups | Contents |
| --- | --- | --- |
| 1 | High-fat milk and cream | Whole milk, cream, flavoured milk |
| 2 | Low-fat milk | Semi-skimmed milk, skimmed milk, soya milk |
| 3 | Yogurts | Yogurt, fromage frais, soya yogurt |
| 4 | Cheese | All types |
| 5 | Butter and animal fat | Butter, spreadable butter, dripping, lard |
| 6 | Margarine and vegetable oils | Margarine (all types), sesame, sunflower, vegetable, and olive oils |
| 7 | Eggs and egg dishes | Boiled, scrambled, poached, fried, omelette, quiche, egg-fried rice |
| 8 | Low-fibre bread | White bread, baguettes, rolls, bagels, muffins, crumpets |
| 9 | High-fibre bread | Brown, soft grain, granary, whole-meal, rye bread, and rolls |
| 10 | Other bread products | Croissants, breadcrumbs, savoury pancakes, Yorkshire pudding, stuffing, dumplings, pastry, naan bread, flour, savoury crackers, and biscuits |
| 11 | High-fibre breakfast cereals | High-fibre cereals (fortified and nonfortified, hot or cold) and porridge |
| 12 | Other breakfast cereals | Low-fibre cereals (fortified and nonfortified, hot or cold) |
| 13 | Rice, pasta, and other grains | Rice (all types), pasta (all types), noodles, couscous, bulgar wheat |
| 14 | Cereal-based mixed meals | Pasta in sauce (canned or homemade), pot noodle |
| 15 | Pizza | Pizza, frozen pizza, French-bread pizza |
| 16 | Biscuits and cakes | Sweet biscuits, cereal bars, cakes (all types), pastries, doughnuts |
| 17 | Puddings | Fruit crumbles, pies, tarts, meringues, cheesecake, custard, instant dessert, jelly, chocolate mousse, rice pudding, sweet pancakes |
| 18 | Ice creams | All ice cream-based desserts |
| 19 | Chocolate and confectionery | Sweets, chewing gum with sugar, marzipan, glacé cherries, candied popcorn, chocolate (plain and filled), icing, sugar, syrups |
| 20 | Sugar-free confectionery | Sugar-free chewing gum, sweets, jelly, and jam |
| 21 | Spreads | Chocolate spread, jam, honey, peanut butter |
| 22 | Meat and poultry | Beef, lamb, pork, chicken, duck (mince, steak, joint, meatballs, chops, spare ribs, belly slices, breast, leg, thigh, and wings) |
| 23 | Meat mixed dishes | Curries, stews, Bolognese, lasagne, pies, casseroles |
| 24 | Processed meat | Bacon, gammon, ham, luncheon meat, corned beef, salami, liver, pate, burgers, sausages, frankfurter, chicken roll |
| 25 | Coated or breaded meat and fish | Fish in batter or breadcrumbs, chicken or turkey in breadcrumbs |
| 26 | Meat substitutes | Soya- or Quorn-based mince, sausages, or burgers |
| 27 | Fish | Baked, poached, grilled, in sauce, steamed, smoked, canned, in paste, in pies |
| 28 | Fried or roast potatoes | Roast potatoes, French fries, oven chips, croquettes, waffles |
| 29 | Boiled or baked potatoes | Boiled, baked, and mashed potatoes; gnocchi |
| 30 | Vegetables (raw or boiled) | All vegetables (raw, pickled, canned, frozen, boiled, or grilled) |
| 31 | Fried vegetables | Fried, stir-fried, or roasted, excluding potatoes |
| 32 | Legumes | Beans, chickpeas, and lentils |
| 33 | Vegetable mixed dishes | Curries, flans, lasagne, pies, in sauce, battered, or breaded |
| 34 | Fresh fruit | All fresh fruit, including canned in juice |
| 35 | Other fruit | Stewed, baked, dried, and canned in syrup |
| 36 | Nuts and seeds | All nuts and seeds, including dried coconut |
| 37 | Crisps and savoury snacks | Crisps (US potato chips), pretzels, tortilla chips, popcorn |
| 38 | Soups | All meat or vegetable soups |
| 39 | Sauces (low energy dense) | Water-, vinegar-, vegetable-, or fruit-based sauces |
| 40 | Sauces (high energy dense) | Egg-, milk-, cream-, cheese-, or oil-based sauces |
| 41 | Condiments | Herbs, spices, and stock cubes |
| 42 | Sugar-sweetened beverages | Fruit squashes or cordials and carbonated drinks with sugar added |
| 43 | Low-energy beverages | Fruit squashes or cordials and carbonated drinks (<10 kcal/100 mL) |
| 44 | Fruit juice | All 100% fruit juices |
| 45 | Hot and powdered drinks | Teas, coffees, hot chocolate or milkshake powder |
| 46 | Water | Mineral, flavoured and tap water |
| 47 | Alcoholic drinks | Beer, alcoholic soda, wine, sherry |

*Reference (9)*
